# Coinfection of Tuberculosis in an Undiagnosed HIV, AIDS Patient Presenting With Shortness of Breath, Constitutional Symptoms and Lymphadenopathy

**DOI:** 10.7759/cureus.15925

**Published:** 2021-06-25

**Authors:** Mirian V Garcia Rivera, Angel Aponte, War War Ko

**Affiliations:** 1 Internal Medicine, St. John's Episcopal Hospital, Far Rockaway, New York, USA

**Keywords:** coinfection, tuberculosis, hiv, opportunistic, infections, aids, antiretroviral, therapy

## Abstract

Tuberculosis (TB) has long been known as an acquired immunodeficiency syndrome (AIDS) defining illness in human immunodeficiency virus (HIV) patients, causing reciprocal advantage for both pathogens throughout the course of the disease, not just constituting a burden for the patient, but also impacting public health globally. We report a case of a 42-year-old man who presented with shortness of breath, generalized lymphadenopathy and weight loss. Subsequently diagnosed with HIV/AIDS and generalized ganglionar TB. Initial computed tomography (CT) of the chest showed extensive mediastinal involvement with large right loculated pleural effusion, with growth of acid-fast bacilli (AFB) on culture. Biopsy of lymph nodes confirmed pathologic changes correlating with *M. tuberculosis *(Caseating granulomatous inflammation), ruling out the possibility of lymphoproliferative disorder. Multiple factors contribute to the immune system decline in AIDS patients, moreover the rapid depletion of Tuberculosis antigen-specific CD4+ T before generalized CD4+T cells. Early assessment for the presence of co-infection and guidance of targeted therapy is critical for management and an important factor in the expected recovery of such patients. Therefore, understanding the pathogenesis of the co-infection, diagnostic approach, possible complications, and the action of concurrent therapy highly active antiretroviral therapy (HAART)/anti-Tuberculosis treatment as well as drug cytotoxicity is paramount.

## Introduction

Tuberculosis (TB) and human immunodeficiency virus (HIV) coinfection is a public health threat. Epidemics of such dangerous diseases as HIV infection and TB continue to increase annually. Tuberculosis has become the main cause of mortality in acquired immunodeficiency syndrome (AIDS) patients. Both diseases have a negative effect on the state of the immune system, affecting the cells of the lymphatic system [1].

Levels of CD4+ T cells have been correlated with the pattern of manifestation of pulmonary TB, finding that those with CD4+ count <200 were more likely to produce atypical patterns [2].

The radiographic manifestations for active TB in HIV-seropositive patients are diverse. The typical presentation of airspace consolidation in the apical or upper zone with or without cavitation is not the most commonly encountered in TB cases. In contrast to this, atypical radiographic findings, including middle/lower zones opacities, mediastinal or hilar lymphadenopathy, pleural effusion, miliary TB, and even normal chest radiographs represent the majority of the reported TB cases. Thus normal chest radiograph does not necessarily rule out TB in HIV infected patients, which sometimes leads to delay in diagnosis [3].

It is a challenge to diagnose and treat co-infected patients with TB and HIV. In this case report we aim to review the diagnostic and treatment approach for both infections.

## Case presentation

The patient is a 42-year-old male with no prior medical history who presented with two weeks of productive cough with yellowish sputum associated with shortness of breath, fatigue, weight loss (about 10 pounds in four weeks), loss of appetite and subjective fever. The patient denied chills, nausea, vomiting, night sweats, recent travel and sick contacts. In addition, the patient noted for the last 4-6 months a swelling of lymph nodes predominantly in the submandibular area. In addition, one month before presentation, he developed a vesicular rash on the left subcostal area spreading to his back. The rash was initially blisters and later became crusted lesions. The patient denied tobacco use although used cannabis regularly and admitted to occasional alcohol use. The patient had 50 female sexual partners with inconsistent barrier protection and denied having a primary care provider for several years.

**Figure 1:**
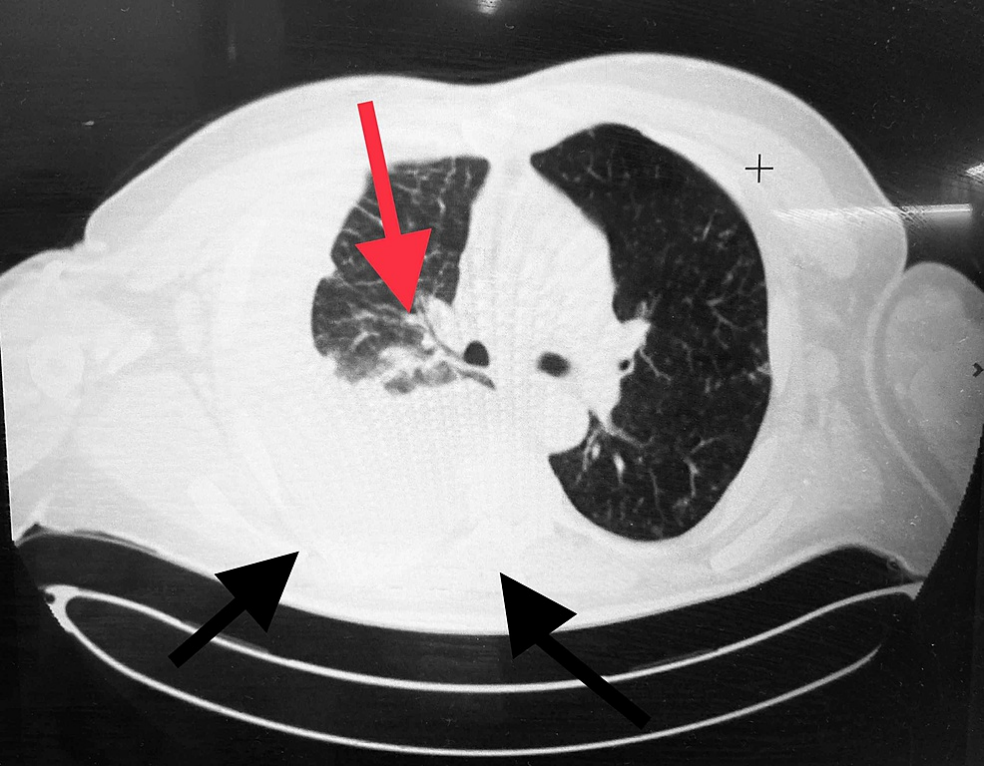
Chest CT with intravenous contrast. Two black arrows showing large loculated right pleural effusion, small cardiac effusion and mediastinal adenopathy (red arrow).

Vital signs at admission were remarkable for temperature of 103.3F, pulse of 132 per minute, respiratory rate of 20. On examination the patient was in mild distress, with diminished breath sounds on right hemithorax, non-tender firm bilateral anterior and posterior cervical lymphadenopathy bilaterally in the supraclavicular and inguinal area. On skin, the patient presented with left-sided crusted lesions along the T5 dermatome region. Laboratory results showed white blood count of 6.9 x 10^9^/L (normal range: 4.5 to 11 x 10^9^/L), serum creatinine of 1.6 mg/dl (normal range: 0.5 to 1.1 mg/dl), whole blood lactic acid of 2.9 mmol/L (normal range: 0.5 to 2.2 mmol/L) and COVID-19 test PCR was negative. Chest X-ray showed a possible pleural-based mass and residual effusion with mass effect on the right lung. The chest CT with contrast showed extensive mediastinal and axillary adenopathy, a large loculated right pleural effusion, patchy bilateral pulmonary infiltrates and a small pericardial effusion. Neck CT showed extensive bilateral lymphadenopathy up to 3 cm, including a necrotic left submandibular node. Adenopathy extended inferiorly through the supraclavicular region and into the chest. The CT of the abdomen and pelvis showed extensive lymphadenopathy. Findings were concerning for lymphoma with differential diagnosis of metastatic disease vs. infectious process (Figure 1).

The patient underwent chest tube placement on the right hemithorax, and 2.4 liters of pleural fluid was drained on the first day. Results of pleural fluid analysis yielded to be exudative in nature, with a high count on lymphocytes, which was suspicious for malignancy or tuberculosis. The patient was placed on airborne isolation precautions.

HIV testing was positive for HIV-1 with CD4 count found on 111 cells/mm^3 ^(normal range: 500 to 1,200 cells/mm^3^), viral load was found on 91,548 copies/ml and the QuantiFERON-TB Gold test was positive.

Urine culture was negative. Four sets of blood cultures were obtained with no growth, three sputum acid-fast bacilli (AFB) smear were negative and culture showed no growth. Pleural fluid culture grew AFB. The left inguinal lymph node was excised and the final biopsy results showed caseating granulomatous inflammation, granulomas were composed of epithelioid histiocytes and occasional multinucleated giant cells, focal caseous necrosis was noted. Additionally, flow cytometry showed no evidence of B-cell or T-cell non-Hodgkin lymphoma. Pulmonary and Infectious Disease (ID) consults recommended to start RIPE (rifampin, isoniazid, pyrazinamide and ethambutol) TB treatment along with bactrim, for pneumocystis pneumonia prophylaxis (PCP).

Infectious Disease recommended to start Highly Active Antiretroviral Therapy (HAART) approximately eight weeks after initiation of TB therapy to avoid immune reconstitution inflammatory syndrome (IRIS).

Ophthalmology was consulted for baseline examination due to treatment with ethambutol. They recommended monitoring kidney function as renal disease could increase risk of ethambutol and isoniazid induced optic neuropathy. Baseline color vision was documented with no current metamorphopsia or scotoma, optic nerve appeared sharp and pink. Follow-up with Ophthalmology was arranged in six months.

The patient continued to have persistent fever. Pleural fluid AFB resulted positive for rare mycobacterium tuberculosis complex by DNA probe. Vancomycin and Azactam were started for broader coverage on top of RIPE therapy with PCP prophylaxis. Since the patient continued to have persistent fever, chest CT was repeated and showed pleural fluid thickening with persistent fluid collection concerning for possible empyema. Repeat procalcitonin downtrended to 0.10 ug/ml (normal range: <0.5 ug/dl) without leukocytosis and subsequently we stopped treatment with Vancomycin and Azactam. The Department of Health (DOH) was informed about the patient's case and recommended to pleural decortication, as such the patient was transferred to a more specialized facility with a TB unit.

## Discussion

Physicians face a great challenge to diagnose, treat and control tuberculosis (TB). Resources and loss of public health capacity, including access to care and maintaining clinical and public health expertise are areas with no sufficient ideal nor clear approach to face these challenges. It is critical to reach those at highest risk for TB, and to identify and implement guidelines to improve testing and treatment. TB rates are higher for some racial and ethnic groups. Like other communities, Blacks/African Americans may face a number of health disparities that contribute to higher rates of TB [4].

African Americans have the most severe burden of HIV of all racial/ethnic groups in the United States and account for a higher proportion of HIV infections at all stages of disease - from new infections to deaths. Blacks/African Americans represent an estimated 53% of all new HIV infections among adults and adolescents (aged 13 years or older) in 2018 and 13% of the U.S. population. Without treatment, as with other opportunistic infections, HIV and TB can work together to shorten the life of the infected patient [5].

Co-infection of TB in HIV infected individuals could be explained by two different mechanisms: either reactivation of latent TB or increase susceptibility for de novo infection with *Mycobacterium tuberculosis* (*M. tuberculosis*). Epidemiological TB studies reviews have shown that HIV status was linked to *M. tuberculosis* clustering, suggesting recent infection as a cause rather than reactivation of latent TB [6].

However, susceptibility for TB infection occurs far before the decrease of CD4+ T-cells count below 500 cells/µL [7], showing that the modifications induced in the immune system by HIV infection that underlies the recognized increased susceptibility to TB go beyond the drop in the cell count.

Multiple factors contribute to the changes in the immune system and progression to AIDS in HIV-infected patients, such as rapid viral replication, increased expression of inflammatory cytokines, and most importantly the loss of CD4+ T cells. Different populations of T cells are preferentially deleted at distinct points during the course of HIV infection. In fact, a selective *M. tuberculosis* antigen-specific CD4+ T cells have been not just identified, but also proved to be depleted before generalized CD4+T cell depletion occurs [8,9].

HAART is associated with decreased incidence of TB. However, despite antiretroviral therapy (ART), the overall incidence rate remains higher (approximately 10-fold) in HIV when compared to non-HIV patients [10].

Some considerations must be taken when starting co-treatment of TB and HIV, medication interaction and development of IRIS among the most important.

The approach to TB testing begins with history and physical exam, to identify the risk factors and clinical criteria that would prompt one to undergo further testing. This includes identifying co-morbidities that put patients at high risk for contracting TB (e.g., HIV), birth country, traveling history, any known contact with people who have TB, high-risk occupation (e.g., healthcare workers), history (hx) of prior positive TB tests, and relevant clinical presentation (e.g., cough greater than two weeks, fever, night sweats, significant weight loss, and lymphadenopathy) [11].

Of note, positive results from the extrapulmonary sites can support the diagnosis of TB, but a negative result cannot rule it out. Patients with risk factors and/or clinical presentations concerning TB should proceed with a screening test to evaluate if they have been exposed to TB, such as tuberculin skin test (TST) or interferon-gamma release assay (IGRA). Currently, there is insufficient data to recommend IGRA over the TST as a first line screening test in adults [11]. Therefore, the history of Bacillus Calmette-Guérin (BCG), the availability and cost of IGRA over the TST should be considered when choosing screening test. Those who are found to have positive screening test results should obtain chest radiograph to evaluate for active pulmonary TB. Those with abnormal chest radiograph (e.g., opacities, cavities, pleural effusions, changes from prior radiographs) should proceed with confirmatory tests, including nucleic acid amplification tests (NAATs), mycobacterial sputum culture, and three sputum acid-fast bacilli (AFB) smears (collected eight to twenty-four hours apart, minimum 3 ml volume, 5-10 ml is optimal volume). Both false-negative and false-positive results are common with AFB smears, and thus, Centers for Disease Control and Prevention (CDC) strongly recommends testing three samples to improve the validity of the results [11,12].

Patients who are immunocompromised or have HIV with CD4 counts <100 cells/mm^3^ should obtain urine mycobacterial culture, serum mycobacterial culture [13], and urine lipoarabinomannan (LAM) lateral flow assay [14, 15] as confirmatory tests in addition to the ones mentioned above. Because the definite diagnosis of TB is established by the identification of *Mycobacterium tuberculosis* from a bodily secretion (e.g., sputum, peritoneal fluid) or biopsy (e.g., pleural, peritoneum, liver, lymph nodes) [16], patients presenting with extrapulmonary TB symptoms who have the specimen taken from those suspected sites should have AFB smear and mycobacterial cultures performed on those specimens.

There are cases of tuberculosis with negative AFB smear and/or culture. In those cases, risk factors, clinical findings, radiographic, laboratory tests, and/or extrapulmonary results may be used to justify starting empiric therapy for patients with high suspicion for TB [17]. Of note, those with a positive screening test that have been excluded to have active TB should be evaluated for latent TB.

There are much evidences that recommend that patients with TB and HIV co-infection should receive therapy of both illnesses regardless of the CD4 cell count level. The goal of treatment of active TB disease in HIV-infected patients is the same as in patient not infected by HIV, although the management of HIV and TB is complex due to drug-drug interactions 18].

Regimen for TB with rifampicin and antiretroviral with efavirenz is the initial choice for treatment of TB/HIV co-infected patients. When using rifabutin in HIV-infected patients on a protease inhibitor-based regimen, the dose should be reduced to 150 mg daily. An adequate collection of patient information should be obtained in order to select optimal regimens for TB/HIV co-infected patients, whenever efavirenz cannot be used and rifabutin is not available. There are significant pharmacokinetic interactions between antiretrovirals and antitubercular drugs, adequate clinical response of both infections can be achieved with an acceptable safety profile when the pharmacological characteristics of drugs are known [18].

Current data compares the treatment outcomes (the benefits and risks) of earlier initiation of ART during TB treatment with the later initiation. The main conclusion of these is that ART needs to be initiated within two to four weeks of starting TB treatment, with the main goal to maximize survival with minimal risks [19].

The risk of IRIS (Immune reconstitution inflammatory syndrome) and CD4 count at the start of ART is higher with a CD4 count of 50 cells/mm^3^. In some studies, patients in earlier ART during TB treatment had 2.19 times more risk of developing TB-associated IRIS than those patients who started ART later during TB treatment irrespective of their CD4 count [19]. It appears that, initiating ART earlier during TB treatment reduces overall mortality and outweighs the risks of TB-IRIS. The reviewed literature showed that earlier initiation of ART in patients with low CD4 reduces mortality though TB-IRIS proportionally increases. It is a critical clinical judgment to determine the importance of initiating ART earlier against the risk of TB-IRIS [19].

This patient clearly has the risk factors and clinical presentations that required workup for TB as well as HIV. TB was high on the differentials given a positive Quantiferon gold test, a positive HIV-1 test, abnormal image findings, and exudative pleural effusion. This patient had three AFB smear collected as per guideline. However, all three AFB smears were negative. As mentioned false negatives are common with AFB smears, therefore, pursuing AFB culture on pleural fluid was the right choice, which helped with the diagnosis and management in this patient. This case could have been improved with sending urine and serum culture for mycobacterium.

Our case patient did not start early ART in 2-8 weeks and was started on late ART at eight weeks as an outpatient.

## Conclusions

African Americans may face a number of challenges that contribute to higher rates of TB, like in the presented case, lack of education and no access to a primary care physician. TB remains a serious threat, especially for people who are infected with human immunodeficiency virus (HIV). Infected HIV patients are more at risk to get opportunistic infections including TB.

Early treatment with ART <8 weeks reduces mortality despite the immune status. This approach should be implemented in future cases of HIV with TB co-infection.
